# Data on NAEP 2011 writing assessment prior computer use

**DOI:** 10.1016/j.dib.2016.07.002

**Published:** 2016-07-06

**Authors:** Tamara P. Tate, Mark Warschauer, Jamal Abedi

**Affiliations:** a University of California, Davis, United States; b University of California, Irvine, United States

## Abstract

This data article contains information based on the 2011 National Assessment of Educational Progress in Writing Restricted-Use Data, available from the National Center for Education Statistics (NCES Pub. No. 2014476). https://nces.ed.gov/nationsreportcard/researchcenter/datatools.aspx. The data include the statistical relationships between survey reports of teachers and students regarding prior use of computers and other technology and writing achievement levels on the 2011 computer-based NAEP writing assessment. This data article accompanies “The Effects of Prior Computer Use on Computer-Based Writing: The 2011 NAEP Writing Assessment” [1].

**Specifications Table**

**Table:** 

Subject area	*Education*
More specific subject area	*Writing*
Type of data	*Tables, Figures*
How data was acquired	*NCES restricted database*
Data format	*Analyzed*
Experimental factors	*Prior access to and use of technology based on survey question responses*
Experimental features	*Jackknife weighting*
Data source location	*USA*
Data accessibility	Data is within this article and available from the National Center for Education Statistics (NCES Pub. No. 2014476). https://nces.ed.gov/nationsreportcard/researchcenter/datatools.aspx

**Value of the data**

• Details of the relationship between 28 survey questions relating to teacher and student use of technology and the 2011 NAEP writing assessment illustrate the outcomes associated with various uses of and access to technology to guide future instruction and investments in resources.
• Models the impact of prior technology use on writing achievement to indicate the positive association between academic use of technology for writing, but not personal or ancillary uses and access.
• Multiple methods used to analyze data to ensure robust understanding of the relationships between access to and use of technology and its potential impact on writing achievement.

## 1 Data

The data in this article models the relationship between students’ reported prior use of and access to computers and their achievement on the first national computer-based writing assessment in the United States, the 2011 National Assessment of Educational Progress (NAEP) assessment. The data models the relationship of survey responses from students and teachers regarding their access to and use of technology for personal and academic uses and students׳ scores on 2 timed writing tasks.

## 2 Experimental design, materials and methods

### 2.1 Details of the survey and assessment

The assessment comprised of a total of 22 writing prompts in three areas, to persuade, to explain, and to convey experience, either real or imagined. Responses were scored by three trained evaluators on a 6-point scale, representing effective skill, adequate skill, developing skill, marginal skill, and little or no skill across three areas of writing – development of ideas, organization of ideas, and language facility and conventions [2], [3]. NAEP evaluators used holistic scoring rubrics to evaluate the response as a whole, rather than assessing independent parts of the response [3]. The scaled booklet-level scores (−2.18 to 3.04) were used as the achievement variable or independent variable for the initial analyses. Additional analysis of student scores was done with the mean of the unscaled scores (interval scale, 1–6) sorting the students and analyzing them by booklet. Variables relating to prior computer use and access included separate student and teacher reported measures of how often (a) the Internet is used to get information, (b) a computer is used for a first draft, (c) a computer is used to make changes in writing, (d) a computer is used to complete writing, (e) a computer is used to write school assignments, (f) a computer is used to write not for school, (g) a computer is used for emails, and (h) a computer is used to write on the Internet. Additionally, self-report measures of teacher use of technology in the classroom were available, providing insight into the degree to which classroom interventions might offset lack of use at home, and teacher professional development relating to technology use. Various demographic groups are included in the data through dichotomous controls for gender, national school lunch eligibility and parental education (as proxies to indicate socioeconomic status), English language learner status (prior, current, or not applicable), students with individualized education plans (IEPs) or 504 plans under the Americans with Disabilities Act, and race/ethnicity.

### 2.2 Structural equation modeling

**Fig. 1:**
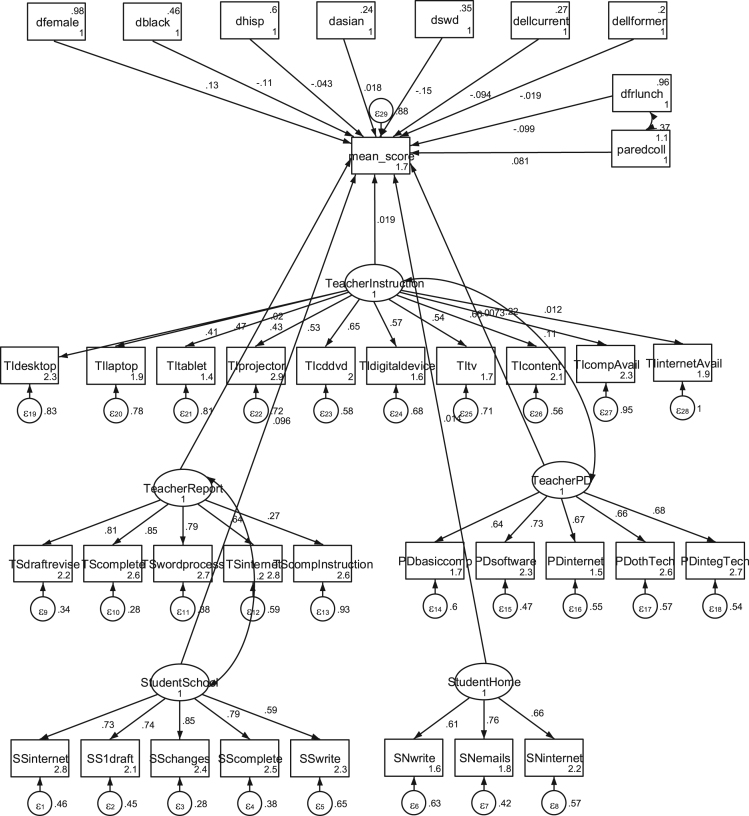
Initial structural equation model showing direct effects of latent variables, with controls, using mean writing score, standardized.

**Fig. 2:**
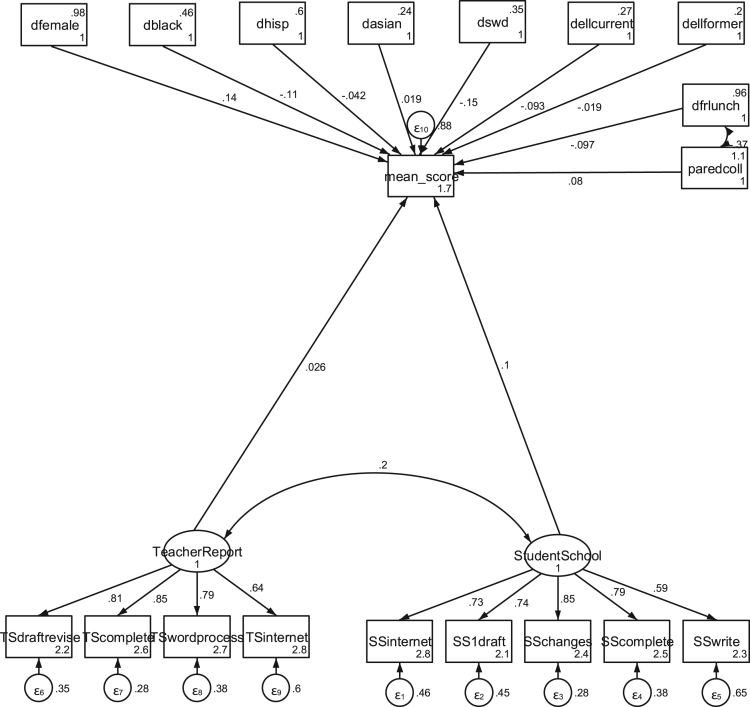
Parsimonious final structural equation model showing direct effects of latent variables on mean writing score, standardized, with controls and no jackknife weighting.

The analysis included structural equation modeling (SEM) of the data using both the IRT scaled scores (“scaled scores”) and the mean of the individual scores by trained reviewers on each essay (“mean scores”) at an aggregate (all essays, regardless of different writing tasks) and booklet-level analysis (isolating each writing) to check for robustness and comparability (Fig. 1, Fig. 2).

### 2.3 Regression

As a robustness check, we also used ordinary least squares (OLS) regression to look at the relationship between reported prior computer use and achievement scores. The regression analysis of the aggregated data can be found in [1]. Following is the analysis of responses by task (the 22 separate writing tasks in the assessment).

### 2.4 Factor analysis

We next used factor analysis to check our latent variable construction. Stata׳s principal factor analysis was used for our confirmatory analysis to check the latent variables we had used in our SEM model. Following are the results from our unrotated factor analysis. The results of the rotated factor analysis can be found in [1] (Table 1, Table 2, Table 3, Table 4, Table 5, Table 6).

## Figures and Tables

**Table 1:** Quartile and other descriptive detail for scaled and mean writing scores outcome variables.

Percentile	Scaled score	Mean score
1%	−1.96	1.00
5%	−1.69	1.00
10%	−1.32	1.50
25%	−0.79	2.00
50%	−0.03	2.50
75%	0.61	3.33
90%	1.17	4.00
95%	1.54	4.50
99%	2.29	5.00
Mean	−0.04	2.64
Standard deviation	0.96	0.98
Variance	0.92	0.96
Skewness	0.14	0.35
Kurtosis	2.66	2.65

**Table 2:** Initial structural equation model loadings. Final loadings can be found in [1].

Mean score analysis						
Latent variable	Coefficient	Standard error	*z*	*P*>|*z*|	95% Confidence	Interval
Student-reported school writing	0.10	0.01	12.78	0.000	0.08	0.11
Student-reported not for school writing	0.01	0.01	2.13	0.033	0.00	0.03
Teacher-reported writing	0.02	0.01	2.76	0.006	0.01	0.03
Teacher instruction	0.02	0.01	2.50	0.013	0.00	0.03
Student-reported and teacher-reported writing (cov)	0.20	0.01	26.61	0.000	0.19	0.21
Teacher PD and teacher instruction (cov)	0.11	0.01	13.35	0.000	0.10	0.13

Scaled score analysis						
Latent variable	Coefficient	Standard error	*z*	*P*>|*z*|	95% Confidence	Interval
Student-reported school writing	0.09	0.01	14.63	0.000	0.08	0.01
Teacher-reported writing	0.07	0.01	11.02	0.000	0.06	0.09
Student-reported and teacher-reported writing (cov)	0.20	0.01	26.64	0.000	0.19	0.21
Teacher PD and teacher instruction (cov)	0.11	0.01	13.35	0.000	0.10	0.13

Teacher professional development latent variable was not statistically significant. RMSEA 0.05 and CFI 0.81.

Student-reported not for school writing, teacher professional development, and teacher instruction latent variables were not statistically significant.

**Table 3:** Correlation matrix of scaled score, mean score, prior use components, and demographic controls in final SEM.

	Scaled score	Mean score	Teacher draft	Teacher complete	Teacher word process	Teacher internet	Student internet	Student 1st draft	Student changes	Student complete	Student write	Female	White
Scaled score	1.00												
Mean score	0.64	1.00											
Teacher draft	0.15	0.09	1.00										
Teacher complete	0.16	0.10	0.70	1.00									
Teacher Word process	0.13	0.09	0.62	0.67	1.00								
Teacher internet	0.07	0.04	0.51	0.51	0.52	1.00							
Student internet	0.23	0.15	0.11	0.12	0.10	0.10	1.00						
Student 1st draft	0.10	0.09	0.12	0.10	0.09	0.08	0.49	1.00					
Student changes	0.22	0.17	0.15	0.16	0.13	0.09	0.55	0.57	1.00				
Student complete	0.26	0.19	0.19	0.20	0.16	0.11	0.51	0.49	0.64	1.00			
Student write	0.11	0.07	0.13	0.13	0.12	0.08	0.37	0.38	0.39	0.38	1.00		
Female	0.25	0.17	−0.00	−0.00	0.00	−0.00	0.08	0.01	0.08	0.05	0.06	1.00	
White	0.26	0.17	0.09	0.12	0.11	0.04	0.07	0.05	0.11	0.14	−0.01	−0.01	1.00
Black	−0.20	−0.14	−0.06	−0.08	−0.06	−0.01	−0.00	−0.01	−0.04	−0.07	0.03	0.01	−0.49
Hispanic	−0.16	−0.10	−0.08	−0.10	−0.08	−0.04	−0.11	−0.07	−0.12	−0.13	−0.05	0.00	−0.59
Asian	0.09	0.06	0.05	0.05	0.02	0.01	0.06	0.05	0.06	0.05	0.06	0.00	−0.24
Other	−0.01	−0.00	0.01	0.01	0.00	0.00	−0.00	−0.00	−0.01	−0.00	0.01	−0.01	−0.06
Free/Red lunch	−0.34	−0.23	−0.16	−0.18	−0.16	−0.07	−0.12	−0.10	−0.18	−0.20	−0.07	0.01	−0.41
Student disability	−0.28	−0.18	−0.02	−0.02	−0.01	−0.02	−0.10	−0.01	−0.05	−0.06	0.01	−0.08	0.00
Current ELL	−0.22	−0.14	−0.06	−0.05	−0.07	−0.03	−0.09	−0.02	−0.07	−0.09	0.00	−0.02	−0.20
Former ELL	−0.05	−0.04	−0.03	−0.04	−0.03	−0.03	−0.03	−0.02	−0.04	−0.06	−0.01	0.00	−0.19
Parent college	0.24	0.16	0.12	0.12	0.09	0.05	0.15	0.12	0.18	0.19	0.12	−0.04	0.21

	Black	Hispanic	Asian	Other	Free/Red lunch	Student disability	Current ELL	Former ELL	Parent college				

Black	1.00												
Hispanic	−0.27	1.00											
Asian	−0.11	−0.13	1.00										
Other	−0.03	−0.03	−0.01	1.00									
Free/Red lunch	0.25	0.27	−0.05	0.01	1.00								
Student disability	0.02	−0.00	−0.05	−0.00	0.07	1.00							
Current ELL	−0.06	0.26	0.06	0.02	0.17	0.03	1.00						
Former ELL	−0.08	0.27	0.04	−0.01	0.13	−0.01	−0.04	1.00					
Parent college	−0.03	−0.26	0.08	−0.01	−0.36	−0.05	−0.11	−0.09	1.00				

**Table 4:** Regression analysis, by writing task, with controls and interactions, using mean writing scores.

	Task 1	Task 2	Task 3	Task 4	Task 5	Task 6	Task 7	Task 8	Task 9	Task 10
Teacher-reported writing	0.01	−0.03	−0.05	−0.03	0.02	0.00	0.00	0.02	0.08**	−0.03
	(0.03)	(0.06)	(0.06)	(0.02)	(0.03)	(0.02)	(0.02)	(0.03)	(0.03)	(0.06)
Student-reported writing	0.14***	0.14*	0.12*	0.09***	0.11***	0.12***	0.05*	0.03	0.00	0.20**
	(0.03)	(0.06)	(0.05)	(0.03)	(0.03)	(0.02)	(0.02)	(0.02)	(0.03)	(0.06)
Female	0.48***	0.72***	0.36***	0.38***	0.43***	0.48***	0.46***	0.41***	0.36***	0.39**
	(0.06)	(0.12)	(0.10)	(0.05)	(0.06)	(0.05)	(0.04)	(0.05)	(0.05)	(0.12)
Black	−0.46***	−0.50**	−0.35*	−0.47***	−0.41***	−0.56***	−0.45***	−0.47***	−0.37***	−0.30
	(0.08)	(0.17)	(0.16)	(0.07)	(0.08)	(0.07)	(0.06)	(0.08)	(0.07)	(0.18)
Hispanic	−0.15	0.03	−0.24	−0.13	−0.16*	−0.31***	−0.15*	−0.18*	−0.06	−0.17
	(0.08)	(0.17)	(0.15)	(0.07)	(0.08)	(0.07)	(0.06)	(0.07)	(0.07)	(0.18)
Asian	0.06	0.16	0.43	0.07	0.50***	0.11	0.13	0.26*	0.42***	0.40
	(0.14)	(0.32)	(0.23)	(0.12)	(0.14)	(0.12)	(0.11)	(0.13)	(0.12)	(0.30)
Other	−0.10	−0.48	−0.83	0.14	−0.30	−0.20	0.23	−0.51	−0.35	−0.81
	(0.47)	(1.42)	(0.88)	(0.33)	(0.51)	(0.35)	(0.31)	(0.44)	(0.74)	(1.25)
Free/Red	−0.28***	−0.32*	−0.22	−0.40***	−0.34***	−0.32***	−0.35***	−0.23***	−0.37***	−0.16
lunch	(0.07)	(0.14)	(0.13)	(0.06)	(0.07)	(0.05)	(0.05)	(0.06)	(0.06)	(0.15)
Parent	0.13*	0.16	0.17	0.21***	0.10	0.22***	0.21***	0.24***	0.18**	0.18
College	(0.06)	(0.13)	(0.12)	(0.06)	(0.06)	(0.05)	(0.05)	(0.06)	(0.06)	(0.14)
Former ELL	0.01	−0.22	−0.22	0.14	−0.21	−0.19	0.01	−0.04	−0.12	−0.28
	(0.21)	(0.40)	(0.29)	(0.16)	(0.16)	(0.13)	(0.12)	(0.15)	(0.15)	(0.41)
Current ELL	−0.67***	0.14	−0.56*	−0.67***	−0.22	−0.66***	−0.80***	−0.76***	−0.86***	−0.83*
	(0.16)	(0.32)	(0.26)	(0.13)	(0.14)	(0.12)	(0.11)	(0.15)	(0.13)	(0.33)
Student w/	−0.77***	−0.83***	−0.27	−0.93***	−0.95***	−0.94***	−0.84***	−0.76***	−0.61***	0.00
Disability	(0.11)	(0.23)	(0.18)	(0.09)	(0.10)	(0.08)	(0.08)	(0.10)	(0.09)	(0.23)
Teacher/	0.01	−0.07	0.01	0.02	−0.02	−0.01	0.02	−0.01	−0.04	0.00
Female	(0.02)	(0.05)	(0.04)	(0.02)	(0.02)	(0.02)	(0.02)	(0.02)	(0.02)	(0.05)
Teacher/	−0.01	−0.02	−0.08	0.01	0.02	0.00	−0.02	−0.01	−0.04	−0.08
Black	(0.03)	(0.06)	(0.06)	(0.03)	(0.03)	(0.03)	(0.02)	(0.03)	(0.03)	(0.06)
Teacher/	0.00	−0.02	0.12	0.02	0.09	0.01	0.00	0.06	0.03	0.03
Asian	(0.05)	(0.13)	(0.11)	(0.06)	(0.05)	(0.04)	(0.06)	(0.05)	(0.05)	(0.11)
Teacher/	−0.02	−0.14*	0.04	0.03	0.03	0.01	0.01	−0.05	−0.06	−0.11
Hispanic	(0.03)	(0.07)	(0.07)	(0.03)	(0.03)	(0.03)	(0.03)	(0.03)	(0.03)	(0.07)
Teacher/	0.00	0.06	0.03	0.03	−0.01	0.00	0.04	0.04	−0.02	−0.03
Free/Red	(0.03)	(0.05)	(0.05)	(0.02)	(0.03)	(0.02)	(0.02)	(0.03)	(0.03)	(0.05)
Teacher/	0.01	0.11*	0.12**	0.01	0.01	0.03	0.00	0.00	−0.01	0.12*
Pt College	(0.03)	(0.05)	(0.05)	(0.02)	(0.03)	(0.02)	(0.02)	(0.03)	(0.02)	(0.05)
Teacher/	−0.02	0.14	0.15	−0.02	−0.18**	0.01	0.00	−0.05	−0.01	0.43***
C ELL	(0.05)	(0.12)	(0.11)	(0.05)	(0.06)	(0.04)	(0.04)	(0.06)	(0.05)	(0.13)
Teacher/	−0.01	0.10	−0.03	−0.24***	−0.02	0.05	−0.02	0.01	0.05	0.18
For ELL	(0.09)	(0.17)	(0.11)	(0.06)	(0.05)	(0.06)	(0.04)	(0.04)	(0.05)	(0.16)
Teacher/	−0.04	0.11	−0.19*	0.03	0.05	0.07*	−0.03	0.05	0.07	−0.24*
St w Dis	(0.04)	(0.08)	(0.08)	(0.04)	(0.04)	(0.03)	(0.03)	(0.04)	(0.04)	(0.10)
Student/	−0.01	0.02	−0.03	−0.01	0.00	−0.04*	0.00	0.01	0.01	−0.15**
Female	(0.02)	(0.05)	(0.04)	(0.02)	(0.02)	(0.02)	(0.02)	(0.02)	(0.02)	(0.05)
Student/	−0.02	−0.01	0.24***	0.04	−0.08**	−0.02	0.01	0.00	−0.02	0.30***
Black	(0.03)	(0.07)	(0.06)	(0.03)	(0.03)	(0.02)	(0.03)	(0.03)	(0.03)	(0.07)
Student/	−0.04	−0.04	−0.03	0.01	−0.08	−0.03	0.06	0.01	−0.08	−0.09
Asian	(0.06)	(0.15)	(0.10)	(0.05)	(0.06)	(0.04)	(0.05)	(0.06)	(0.05)	(0.13)
Student/	−0.03	−0.09	−0.05	0.02	−0.02	−0.01	0.04	0.01	0.02	−0.12
Hispanic	(0.03)	(0.07)	(0.06)	(0.03)	(0.03)	(0.02)	(0.02)	(0.03)	(0.03)	(0.07)
Student/	−0.08**	−0.08	0.02	−0.08***	−0.01	−0.04*	−0.05*	0.01	0.01	−0.05
Free/Red	(0.03)	(0.05)	(0.05)	(0.02)	(0.02)	(0.02)	(0.02)	(0.03)	(0.02)	(0.06)
Student/	−0.04	−0.09	−0.14**	0.01	−0.05*	−0.03	0.02	0.05*	0.06**	−0.12*
Pt College	(0.03)	(0.05)	(0.05)	(0.02)	(0.02)	(0.02)	(0.02)	(0.02)	(0.02)	(0.06)
Student/	0.03	0.38***	−0.13	−0.05	0.34***	−0.03	−0.08*	0.04	−0.02	−0.29**
C ELL	(0.06)	(0.09)	(0.10)	(0.05)	(0.04)	(0.04)	(0.03)	(0.05)	(0.04)	(0.11)
Student/	0.16	0.20	−0.04	−0.08	−0.01	−0.02	−0.08	−0.06	0.00	−0.01
For ELL	(0.08)	(0.15)	(0.11)	(0.06)	(0.06)	(0.05)	(0.05)	(0.05)	(0.05)	(0.14)
Student/	−0.03	−0.19*	0.25***	−0.03	−0.12***	−0.08**	−0.04	−0.09*	−0.08**	0.48***
St w Dis	(0.03)	(0.07)	(0.06)	(0.03)	(0.03)	(0.03)	(0.03)	(0.04)	(0.03)	(0.08)
Constant	2.59***	2.62***	2.77***	2.84***	2.77***	2.76***	2.69***	2.54***	2.60***	2.68***
	(0.07)	(0.15)	(0.13)	(0.06)	(0.07)	(0.05)	(0.05)	(0.07)	(0.06)	(0.15)
*N*	1680	1700	1660	1660	1690	1660	1670	1670	1710	1660
R-sq	0.21	0.07	0.10	0.25	0.28	0.32	0.32	0.21	0.24	0.10

	Task 11	Task 12	Task 13	Task 14	Task 15	Task 16	Task 17	Task 18	Task 19	Task 20	Task 21	Task 22
Teacher-reported writing	0.00	0.00	0.01	0.01	0.03	0.02	0.08**	−0.03	0.02	0.00	−0.01	0.02
	(0.02)	(0.02)	(0.03)	(0.02)	(0.04)	(0.03)	(0.03)	(0.03)	(0.03)	(0.03)	(0.03)	(0.02)
Student-reported writing	0.12***	0.07**	0.12**	0.10***	0.04	0.08**	−0.02	0.10***	0.08*	0.09**	0.08**	0.07***
	(0.02)	(0.02)	(0.04)	(0.02)	(0.04)	(0.03)	(0.03)	(0.03)	(0.03)	(0.03)	(0.03)	(0.02)
Female	0.46***	0.39***	0.55***	0.44***	0.47***	0.44***	0.40***	0.34***	0.45***	0.48***	0.45***	0.47***
	(0.04)	(0.04)	(0.07)	(0.04)	(0.09)	(0.05)	(0.05)	(0.05)	(0.06)	(0.06)	(0.06)	(0.04)
Black	−0.48***	−0.44***	−0.51***	−0.49***	−0.44***	−0.51***	−0.44***	−0.50***	−0.51***	−0.51***	−0.47***	−0.39***
	(0.06)	(0.06)	(0.11)	(0.06)	(0.13)	(0.08)	(0.07)	(0.08)	(0.09)	(0.09)	(0.08)	(0.06)
Hispanic	−0.24***	−0.17**	−0.14	−0.27***	−0.08	−0.16*	−0.17*	−0.19*	−0.06	−0.08	−0.15	−0.18**
	(0.06)	(0.06)	(0.11)	(0.06)	(0.12)	(0.08)	(0.07)	(0.08)	(0.08)	(0.08)	(0.08)	(0.06)
Asian	−0.02	0.19	−0.06	0.17	0.10	0.16	0.11	0.06	0.14	0.30*	−0.01	−0.04
	(0.10)	(0.10)	(0.18)	(0.11)	(0.23)	(0.13)	(0.12)	(0.15)	(0.15)	(0.14)	(0.14)	(0.10)
Other	0.04	−0.35	0.07	−0.01	−0.40	−0.11	−0.05	0.04	0.17	−0.44	0.20	0.64
	(0.27)	(0.38)	(0.45)	(0.43)	(0.89)	(0.45)	(0.44)	(0.39)	(0.52)	(0.36)	(0.36)	(0.60)
Free/Red	−0.35***	−0.33***	−0.31***	−0.26***	−0.27*	−0.36***	−0.42***	−0.32***	−0.50***	−0.32***	−0.25***	−0.33***
Lunch	(0.05)	(0.05)	(0.09)	(0.05)	(0.11)	(0.06)	(0.06)	(0.06)	(0.07)	(0.07)	(0.07)	(0.05)
Parent	0.15**	0.19***	0.09	0.24***	0.15	0.19**	0.14*	0.16**	0.17**	0.30***	0.14*	0.17***
College	(0.05)	(0.05)	(0.08)	(0.05)	(0.10)	(0.06)	(0.06)	(0.06)	(0.06)	(0.07)	(0.06)	(0.05)
Former ELL	−0.23	0.01	−0.20	0.02	−0.18	−0.21	0.03	0.05	−0.30	−0.19	−0.22	−0.01
	(0.12)	(0.11)	(0.24)	(0.11)	(0.28)	(0.17)	(0.14)	(0.16)	(0.15)	(0.17)	(0.16)	(0.12)
Current ELL	−0.90***	−0.70***	−0.90***	−0.64***	0.37	−0.42**	−0.85***	−0.83***	−0.97***	−0.57**	−0.88***	−0.86***
	(0.12)	(0.11)	(0.22)	(0.11)	(0.22)	(0.14)	(0.12)	(0.16)	(0.14)	(0.19)	(0.15)	(0.11)
Student w/	−0.80***	−0.90***	−0.48***	−0.87***	−1.04***	−0.90***	−0.76***	−0.78***	−0.91***	−0.60***	−0.49***	−0.81***
Disability	(0.07)	(0.07)	(0.14)	(0.07)	(0.16)	(0.10)	(0.09)	(0.10)	(0.09)	(0.11)	(0.10)	(0.07)
Teacher/	−0.03	0.02	0.03	−0.03	−0.05	−0.03	−0.03	0.03	−0.04	0.01	0.01	−0.01
Female	(0.02)	(0.02)	(0.03)	(0.02)	(0.03)	(0.02)	(0.02)	(0.02)	(0.02)	(0.02)	(0.02)	(0.02)
Teacher/	−0.01	0.02	0.01	0.00	−0.01	0.02	−0.04	0.03	0.02	−0.01	−0.04	−0.01
Black	(0.02)	(0.02)	(0.04)	(0.02)	(0.05)	(0.03)	(0.03)	(0.03)	(0.03)	(0.03)	(0.03)	(0.02)
Teacher/	0.03	0.03	0.04	0.04	0.05	0.14**	−0.02	0.05	−0.05	0.01	0.01	0.01
Asian	(0.04)	(0.05)	(0.08)	(0.04)	(0.07)	(0.05)	(0.05)	(0.06)	(0.05)	(0.05)	(0.07)	(0.05)
Teacher/	0.01	0.02	−0.01	0.02	−0.07	0.02	0.00	0.03	0.00	−0.02	0.00	−0.02
Hispanic	(0.02)	(0.02)	(0.04)	(0.02)	(0.05)	(0.03)	(0.03)	(0.03)	(0.04)	(0.03)	(0.03)	(0.03)
Teacher/	0.05**	−0.03	−0.02	0.00	0.03	−0.02	−0.06*	0.03	0.04	−0.01	0.06*	−0.01
Free/Red	(0.02)	(0.02)	(0.03)	(0.02)	(0.04)	(0.03)	(0.02)	(0.03)	(0.03)	(0.03)	(0.03)	(0.02)
Teacher/	0.03	0.03	0.02	0.03	0.04	0.00	−0.02	0.02	0.03	0.02	0.03	0.03
Pt College	(0.02)	(0.02)	(0.03)	(0.02)	(0.04)	(0.03)	(0.02)	(0.02)	(0.03)	(0.03)	(0.03)	(0.02)
Teacher/	0.02	−0.01	−0.04	0.01	0.00	−0.16**	−0.06	−0.07	0.00	−0.01	0.02	−0.06
C ELL	(0.04)	(0.04)	(0.08)	(0.04)	(0.08)	(0.05)	(0.04)	(0.06)	(0.06)	(0.07)	(0.06)	(0.04)
Teacher/	−0.05	−0.04	0.10	0.03	−0.01	0.00	−0.01	−0.06	−0.04	0.00	−0.04	0.05
For ELL	(0.04)	(0.04)	(0.11)	(0.05)	(0.11)	(0.07)	(0.04)	(0.05)	(0.06)	(0.08)	(0.06)	(0.06)
Teacher/	−0.02	0.03	0.04	0.01	−0.04	0.05	0.06*	0.03	−0.04	0.08	−0.03	−0.01
St w Dis	(0.03)	(0.03)	(0.05)	(0.02)	(0.06)	(0.04)	(0.03)	(0.04)	(0.03)	(0.04)	(0.03)	(0.03)
Student/	−0.01	−0.01	0.01	0.00	0.08*	0.04	0.01	−0.03	0.00	−0.03	0.01	0.01
Female	(0.02)	(0.02)	(0.03)	(0.02)	(0.03)	(0.02)	(0.02)	(0.02)	(0.02)	(0.02)	(0.02)	(0.02)
Student/	−0.05*	−0.02	−0.04	−0.01	−0.03	−0.06*	−0.02	0.00	−0.04	0.00	−0.01	−0.01
Black	(0.02)	(0.02)	(0.04)	(0.02)	(0.05)	(0.03)	(0.03)	(0.03)	(0.03)	(0.04)	(0.03)	(0.02)
Student/	−0.05	0.04	0.06	−0.04	−0.06	−0.08	0.03	0.07	0.00	0.00	0.07	0.05
Asian	(0.04)	(0.04)	(0.08)	(0.04)	(0.11)	(0.06)	(0.05)	(0.06)	(0.07)	(0.06)	(0.06)	(0.05)
Student/	−0.03	−0.03	−0.02	−0.01	0.05	−0.02	0.04	0.01	−0.06	−0.01	0.01	0.03
Hispanic	(0.02)	(0.02)	(0.05)	(0.02)	(0.05)	(0.03)	(0.03)	(0.03)	(0.03)	(0.03)	(0.03)	(0.02)
Student/	−0.07***	0.01	−0.04	−0.07***	0.01	−0.04	0.04	−0.05*	−0.03	0.00	−0.05*	−0.05**
Free/Red	(0.02)	(0.02)	(0.03)	(0.02)	(0.04)	(0.02)	(0.02)	(0.02)	(0.03)	(0.03)	(0.02)	(0.02)
Student/	0.01	0.01	−0.06	−0.02	−0.03	−0.01	0.09***	0.02	0.05	0.00	−0.01	−0.01
Pt College	(0.02)	(0.02)	(0.03)	(0.02)	(0.04)	(0.02)	(0.02)	(0.02)	(0.03)	(0.03)	(0.02)	(0.02)
Student/	−0.09*	−0.01	−0.08	0.00	0.34***	0.38***	−0.09*	−0.06	0.01	0.05	−0.05	−0.10**
C ELL	(0.04)	(0.04)	(0.07)	(0.04)	(0.07)	(0.04)	(0.04)	(0.06)	(0.05)	(0.07)	(0.05)	(0.04)
Student/	0.08	0.03	−0.07	0.03	−0.10	0.06	−0.02	−0.04	0.05	−0.04	0.02	−0.06
For ELL	(0.05)	(0.05)	(0.09)	(0.05)	(0.10)	(0.06)	(0.05)	(0.06)	(0.06)	(0.07)	(0.07)	(0.05)
Student/	0.00	−0.08**	−0.14**	−0.02	−0.07	−0.09*	−0.12***	−0.07*	−0.04	−0.09	−0.07*	−0.04
St w Dis	(0.03)	(0.02)	(0.05)	(0.02)	(0.05)	(0.04)	(0.03)	(0.03)	(0.04)	(0.05)	(0.03)	(0.02)
Constant	2.73***	2.72***	2.77***	2.57***	2.61***	2.69***	2.68***	2.72***	2.78***	2.71***	2.68***	2.60***
	(0.05)	(0.05)	(0.09)	(0.05)	(0.11)	(0.07)	(0.06)	(0.07)	(0.07)	(0.07)	(0.07)	(0.05)
*N*	1660	1620	1630	1690	1670	1640	1640	1640	1650	1680	1690	1700
R-sq	0.37	0.34	0.13	0.34	0.12	0.30	0.27	0.21	0.29	0.20	0.18	0.32

*N* rounded to the nearest 10. Standard errors in parentheses.

* *p*<0.05.

** *p*<0.01.

*** *p*<0.001.

**Table 5:** Eigenvalues for principal factor analysis (unrotated) of computer use in the NAEP teacher and student surveys.

Factor	Eigenvalue	Difference	Proportion	Cumulative
**Factor 1**	**3.93**	**0.78**	**0.38**	**0.38**
**Factor 2**	**3.15**	**0.94**	**0.30**	**0.68**
**Factor 3**	**2.21**	**0.70**	**0.21**	**0.89**
**Factor 4**	**1.51**	**0.84**	**0.14**	**1.03**
Factor 5	0.67	0.08	0.06	1.10
Factor 6	0.59	0.34	0.06	1.16
Factor 7	0.25	0.14	0.02	1.18
Factor 8	0.11	0.02	0.01	1.19
Factor 9	0.08	0.05	0.01	1.20
Factor 10	0.03	0.03	0.00	1.20
Factor 11	−0.00	0.01	−0.00	1.20
Factor 12	−0.02	0.03	−0.00	1.20
Factor 13	−0.04	0.01	−0.00	1.19
Factor 14	−0.05	0.02	−0.00	1.19
Factor 15	−0.07	0.00	−0.01	1.18
Factor 16	−0.07	0.01	−0.01	1.18
Factor 17	−0.08	0.03	−0.01	1.17
Factor 18	−0.11	0.01	−0.01	1.16
Factor 19	−0.12	0.01	−0.01	1.15
Factor 20	−0.14	0.01	−0.01	1.14
Factor 21	−0.14	0.01	−0.01	1.12
Factor 22	−0.15	0.00	−0.01	1.11
Factor 23	−0.15	0.02	−0.01	1.09
Factor 24	−0.17	0.01	−0.02	1.08
Factor 25	−0.18	0.01	−0.02	1.06
Factor 26	−0.19	0.02	−0.02	1.04
Factor 27	−0.21	0.00	−0.02	1.02
Factor 28	−0.21	.	0.02	1.00

LR test: independent vs. saturated: chi^2^ (378)=2.0*e*+05 Prob>chi^2^=0.0000

Factor analysis/correlation; number of observations=22,150; method: principal factors

Retained factors=10 Rotation: (unrotated) Number of parameters=235.

**Table 6:** Factor loadings (pattern matrix) and unique variances for 28 student and teacher survey questions relating to writing with computers.

Variable	**Factor 1**	**Factor 2**	**Factor 3**	**Factor 4**	Factor 5	Factor 6	Factor 7	Factor 8	Factor 9	Factor 10	Uniqueness
Student-reported											
											
School-related use											
Internet	**0.40**	**0.57**	0.02	0.03	−0.17	0.065	0.01	−0.02	0.03	−0.02	0.47
1st draft	**0.40**	**0.58**	0.02	0.04	−0.19	0.05	0.00	0.01	−0.00	−0.03	0.46
Changes	**0.45**	**0.62**	0.02	−0.01	−0.28	0.06	−0.01	−0.00	0.00	0.2	0.33
Complete	**0.44**	**0.58**	0.01	−0.05	−0.24	0.06	−0.01	0.00	0.00	0.03	0.40
Write	0.39	**0.53**	0.02	0.08	0.11	−0.02	−0.01	0.01	−0.02	−0.02	0.54
											
Home use											
Write	0.23	**0.42**	0.04	0.21	0.34	−0.06	0.01	−0.01	0.00	−0.01	0.60
Emails	0.26	**0.44**	0.03	0.20	0.38	−0.10	0.01	0.01	−0.01	0.01	0.54
Internet	0.24	**0.41**	0.03	0.19	0.34	−0.08	0.01	−0.02	−0.00	0.02	0.62
											
Teacher-reported											
											
Instructional uses											
Desktop	0.35	−0.23	−0.05	0.08	−0.03	0.03	0.11	−0.16	−0.09	0.05	0.77
Laptop	0.36	−0.22	−0.07	0.22	−0.01	0.03	−0.15	0.18	0.02	−0.03	0.71
Tablet	0.28	−0.23	0.02	0.21	0.03	0.19	0.06	0.04	0.08	0.09	0.77
Projector	**0.40**	−0.31	−0.12	0.36	−0.13	−0.31	−0.08	−0.02	0.07	0.03	0.47
Cd/dvd	**0.43**	−0.34	−0.01	0.29	−0.04	0.09	−0.04	−0.09	−0.06	−0.03	0.60
Digital device	0.38	−0.28	−0.01	0.23	0.02	0.27	0.05	0.02	0.02	0.01	0.64
TV	0.37	−0.28	0.03	0.20	0.06	0.32	0.11	0.02	−0.02	−0.00	0.63
Content	**0.47**	−0.33	−0.07	0.30	−0.03	−0.01	0.03	−0.01	−0.06	−0.07	0.56
Comp available	0.26	−0.11	−0.01	−0.05	−0.02	−0.06	0.02	0.16	−0.15	0.03	0.86
Internet	0.02	−0.02	−0.00	−0.08	0.11	0.27	0.01	0.00	0.13	−0.01	0.89
											
Student use											
Draft/revise	**0.61**	−0.18	−0.11	−**0.46**	0.06	−0.07	0.00	0.04	−0.04	0.01	0.36
Complete	**0.60**	−0.17	−0.15	−**0.50**	0.08	−0.05	−0.01	−0.01	0.01	0.03	0.33
Word process	**0.56**	−0.17	−0.13	−**0.49**	0.09	−0.05	−0.03	−0.02	0.04	0.00	0.39
Internet	**0.56**	−0.21	−0.10	−0.30	0.09	0.06	0.02	−0.04	0.04	−0.08	0.55
Computer instruct	**0.46**	−0.33	−0.14	0.28	−0.09	−0.28	−0.07	−0.03	0.07	0.01	0.48
											
Professional Dev											
Basic comp	0.09	−0.09	**0.63**	−0.05	0.05	0.14	−0.21	−0.05	−0.03	0.01	0.51
Software	0.12	−0.09	**0.68**	−0.03	−0.02	−0.05	−0.03	0.02	0.01	−0.00	0.51
Internet	0.11	−0.08	**0.66**	−0.05	0.02	0.06	−0.22	−0.03	−0.01	0.02	0.49
Other tech	0.16	−0.11	**0.62**	−0.02	−0.04	−0.15	0.21	0.02	0.03	−0.02	0.51
Integr tech	0.14	−0.09	**0.65**	−0.03	−0.04	−0.15	0.21	0.04	0.02	−0.01	0.48
