# Supplementary material for: Data on NAEP 2011 writing assessment prior computer use
Source: Data Brief. 2016 Jul 6;8:978–89. doi: 10.1016/j.dib.2016.07.002 (PMC4961279; doi:10.1016/j.dib.2016.07.002)
Supplement: Supplementary file 1 — Supplementary material [file mmc1.doc]

| UNIVERSITY OF CALIFORNIA, IRVINE  BERKELEY • DAVIS • IRVINE • LOS ANGELES • MERCED • SAN DIEGO • SAN FRANCISCO | 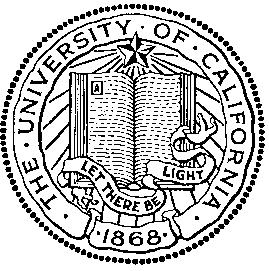 | SANTA BARBARA • SANTA CRUZ |
| --- | --- | --- |

| Department of Education | 3200 Education  Irvine, CA 92697-5500  (949) 431-6540  www.gse.uci.edu |
| --- | --- |

June 23, 2016

Ladies and Gentlemen::

We are pleased to submit for your review our revised Data in Brief article “***Data on NAEP 2011 Writing Assessment Prior Computer Use.”***  We confirm that none of the authors have any conflict of interest to disclose.

Thank you for your consideration of our work.

Sincerely,

Tamara Powell Tate, JD

Doctoral Student

School of Education
University of California, Irvine
